# Fibrolipoma of the Buccal Mucosa: A Case Report and Review of the Literature

**DOI:** 10.1155/2016/5060964

**Published:** 2016-01-17

**Authors:** Masayasu Iwase, Naotaka Saida, Yoko Tanaka

**Affiliations:** Department of Dentistry and Oral Surgery, Hakujikai Memorial General Hospital, 5-11-1 Shikahama, Adachi-ku, Tokyo 123-0864, Japan

## Abstract

Lipomas are common benign soft tissue neoplasms derived from mature adipose tissue. However, they rarely arise in the oral cavity. Fibrolipoma is a histological variant of lipoma that mainly affects the buccal mucosa and causes functional and cosmetic issues. This article describes the case of a 71-year-old male with a fibrolipoma of the left buccal mucosa and a review of previous articles about fibrolipoma.

## 1. Introduction

Lipomas are common benign neoplasms derived from adipose tissue. The etiology of lipomas is uncertain. They mainly affect the trunk region, shoulders, upper arms, and neck [1], and lipomas of the oral cavity are rare, accounting for 1 to 4% of benign oral tissue tumors [2, 3]. Oral lipomas can occur at various sites including the major salivary glands, buccal mucosa, tongue, lips, palate, vestibule, and the floor of the mouth. A number of case reports have described cases in which lipoma or variants of lipoma arose in various oral locations [3, 4]. Histologically, lipomas can be classified into classic lipoma and variant forms of lipoma, such as fibrolipomas, spindle lipomas, intramuscular lipomas, angiolipomas, salivary gland lipomas, pleomorphic lipomas, myxoid lipomas, and atypical lipomas [1, 3, 4]. There have only been a few reports about fibrolipoma of the oral cavity [5–8]. We describe the case of a patient with fibrolipoma of the buccal mucosa.

## 2. Case Presentation

**Figure 1:**
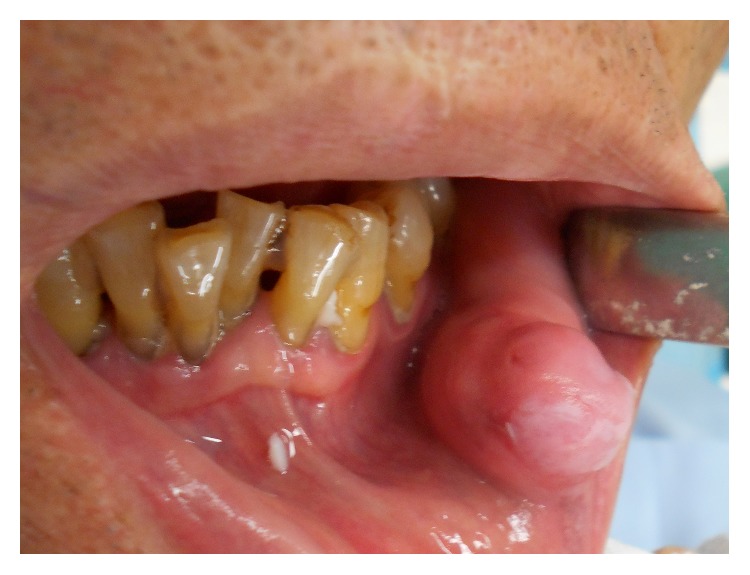
Preoperative intraoral view showing a solitary swelling in the buccal mucosa.

**Figure 2:**
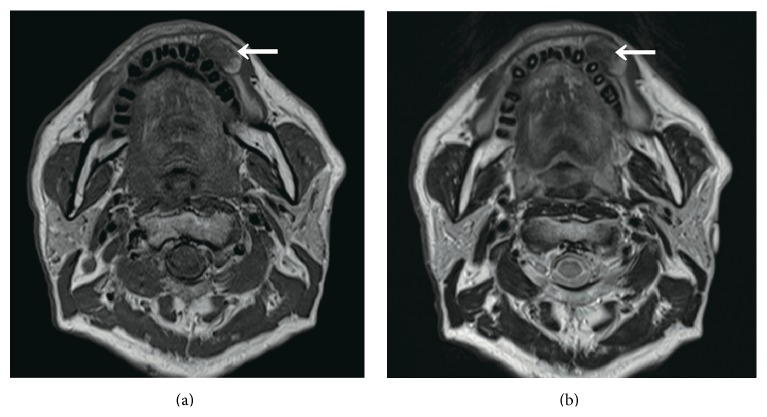
MRI findings of the lesion. MRI showed a well-defined mass that exhibited low signal intensity on both (a) T_1_- and (b) T_2_-weighted images.

**Figure 3:**
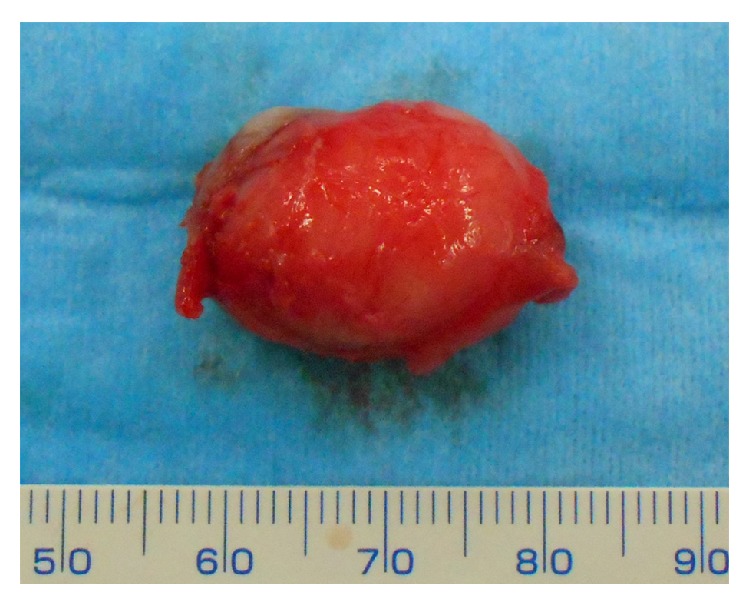
Gross appearance of the lesion. The excised specimen was pinkish in color, displayed a soft consistency, and measured 20 × 15 mm in size.

**Figure 4:**
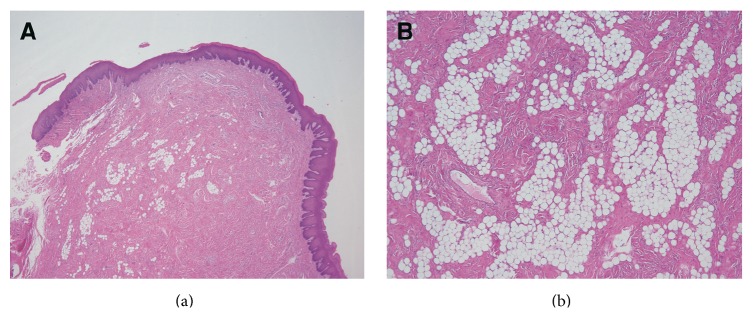
Histological findings of the lesion. (a) A histological examination showed that the lesion contained an overlying epithelium and adipose tissue within dense collagen fibers (hematoxylin and eosin stain, ×10) and (b) mature adipose tissue interspersed by broad bands of dense connective tissue (×40).

A 71-year-old male patient visited our hospital with a chief complaint of swelling of the left buccal mucosa. The swelling had first been noticed two years earlier and had subsequently exhibited gradual continuous enlargement. The patient had bitten the swollen region of his buccal mucosa several times, causing bleeding and pain. An intraoral examination revealed a pinkish, ill-defined oval swelling in the left buccal mucosa (Figure 1). On palpation, the swelling was mainly soft but firm in parts; nonfluctuant; and mobile, and its margins were unclear. The firm regions were composed of scar tissue. The provisional diagnosis was lipoma. A magnetic resonance imaging (MRI) scan of the lesion showed well-defined borders and low signal intensity on both T_1_- and T_2_-weighted images (Figure 2). The lesion was excised under local anesthesia. The excised specimen appeared to be encapsulated, soft, and pinkish in color and measured 25 × 15 mm (Figure 3). During a histopathological examination, the lesion was found to have an overlying epithelium and to be composed of mature adipose tissue within dense collagen fibers (Figure 4). As a result, it was histologically classified as a fibrolipoma. The patient's postoperative course was uneventful. No recurrence of the lesion has been observed after 6 months.

## 3. Discussion

Fibrolipoma is a histological variant of lipoma characterized by a significant fibrous component intermixed with lobules of adipose tissue [1, 5, 8]. The consistency of such lesions varies from soft to firm, depending on the quantity and distribution of fibrous tissue and the depth of the tumor [1, 5]. Fibrolipomas most commonly arise in the buccal mucosa, followed by the tongue, which is also true for classic lipomas [3, 5]. Oral lipoma and its variants have been reported to occur in all age groups but are most frequently seen in patients ranging in age from 40 to 60 years [3, 4, 9, 10]. Previous studies have reported that lesions in the oral cavity exhibit a mean diameter of 2 cm [3, 4]. Therefore, the present case did not involve any particularly unusual clinical findings.

In large case series studies of lipoma and variants of lipoma of the oral cavity, it was found that fibrolipoma accounted for 1.6% [4] and 8.3% [9] of lesions, respectively. However, other similar case series studies have reported a very high incidence of fibrolipoma [3, 10]. These discrepancies cannot be explained by racial or geographic characteristics. As the classification of lipomas depends on the quantity of fibrous tissue present, the diagnosis of such lesions relies on pathologists' judgments. To the best of our knowledge, about 40 cases of fibrolipoma of the oral cavity are described in the English literature [5–8].

The etiopathogeneses of lipoma and fibrolipoma remain unknown. A previous study suggested that fibrolipoma (1) is a congenital lesion caused by an endocrinal imbalance, (2) arises via the degeneration of a fibromatous tumor, or (3) arises from the maturation of lipoblastomatosis [11]. On the other hand, another study described a fibrolipoma that formed beneath a complete denture [12]. It has also been suggested that repeated mild trauma can trigger the proliferation of fatty tissue [2]. We consider that the present case involved a classic lipoma and hyperplasia of fibrous tissue caused by repeated chewing-related trauma.

MRI has been reported to be useful for diagnosing lipomatous lesions of the oral cavity [1, 13]. Lipomas generally display high signal intensity and appear to be well-encapsulated masses on both T_1_- and T_2_-weighted images [1, 14]. However, in the present case the lesion demonstrated low signal intensity on MRI. It is possible that the fibroma-like findings exhibited by the lesion on MRI were due to the fact that it contained abundant collagenous fibers. The nonadipose components of lipomas have been found to display decreased signal intensity on all pulse sequences [1].

Lipoma is mainly treated by surgical excision [1]. The prognosis of lipoma is generally favorable, and recurrence is unlikely when surgery is performed appropriately. However, a case in which a lipoma of the buccal mucosa, which was diagnosed by biopsy, underwent transformation to liposarcoma has been reported [15]. In a previous study, the proliferative activity of lipomas was examined by immunohistochemically analyzing the expression of proliferating cell nuclear antigen and Ki-67. As a result, it was suggested that Ki-67 expression is indicative of recurrence or malignant transformation [9]. Another study found that fibrolipoma exhibits higher Ki-67 expression than classic lipoma and other variants of lipoma [3]. The present patient should be examined for malignant changes during the follow-up period.
